# Ameliorative role of SIRT1 in peritoneal fibrosis: an in vivo and in vitro study

**DOI:** 10.1186/s13578-021-00591-8

**Published:** 2021-04-27

**Authors:** Yanhong Guo, Liuwei Wang, Rong Gou, Yulin Wang, Xiujie Shi, Yage Zhang, Xinxin Pang, Lin Tang

**Affiliations:** 1 Department of Nephropathy, The First Affiliated Hospital of Zhengzhou University, No. 1 East Jianshe Road, Zhengzhou, 450052 Henan China; 2 Department of Nephropathy, Henan Provincial Hospital of Traditional Chinese Medicine (The Second Hospital Affiliated to Henan University of Chinese Medicine), NO. 6, Dongfeng Road, Jinshui District, Zhengzhou, 450002 Henan China

**Keywords:** Peritoneal fibrosis, Peritoneum, SIRT1, TGF‐β, Mesothelial-mesenchymal transition

## Abstract

**Background:**

Peritoneal fibrosis is one of the major complications induced by peritoneal dialysis (PD). Damaged integrity and function of peritoneum caused by peritoneal fibrosis not only limits the curative efficacy of PD and but affects the prognosis of patients. However, the detailed mechanisms underlying the process remain unclear and therapeutic strategy targeting TGF‐β is deficient. Transforming growth factor‐β (TGF‐β) signaling participates in the progression of peritoneal fibrosis through enhancing mesothelial-mesenchymal transition of mesothelial cells.

**Methods:**

The study aims to demonstrate the regulatory role of Sirtuin1 (SIRT1) to the TGF‐β signaling mediated peritoneal fibrosis. SIRT1^−/−^ mice were used to establish animal model. Masson’s staining and peritoneal equilibration assay were performed to evaluate the degree of peritoneal fibrosis. QRT-PCR assays were used to estimate the RNA levels of Sirt1 and matrix genes related to peritoneal fibrosis, and their protein levels were examined by Western blot assays.

**Results:**

SIRT1 significantly decreased in vivo post PD treatment. SIRT1 knockout exacerbated peritoneal fibrosis both in vivo and vitro. Overexpression of SIRT1 efficiently inhibited peritoneal fibrosis by inhibiting the peritoneal inflammation and the activation of TGF‐β signaling.

**Conclusion:**

SIRT1 ameliorated peritoneal fibrosis both in vivo and in vitro through inhibiting the expression of protein matrix induced by TGF‐β signaling.

## Background

Peritoneal dialysis (PD) has been widely used for both acute kidney injury and chronic renal failure [1]. However, peritoneal fibrosis induced by long-term PD has been a devastating actuality in clinic, which significantly affects the prognosis of patients [2–4]. Peritoneal fibrosis is generally defined as the morphologic and functional abnormality of the peritoneal membrane (PM) [5]. The progressive decrease of mesothelial cells (MCs) affects the integrity and function of the mesothelial monolayer, and the deposition of matrix affects the structure of sub-mesothelium layer [6, 7]. Thus, severe peritoneal fibrosis leads to marked dysregulation of both ultrafiltration and diffusive functions of PM [8].

Although accumulating evidence has demonstrated that composition in bioincompatible PD solutions, including glucose, glucose degradation products (GDP) and advanced glycation end products (AGEs), affects the normal physiological functions of MCs and enhances the mesothelial-mesenchymal transition [9, 10], the detailed mechanisms underlying this process remain unclear. The role of transforming growth factor‐β (TGF‐β) during the progression of peritoneal fibrosis has drawn great attention in recent years, and TGF‐β has been identified as a significant contributor to fibrosis development during PD [11]. It has been reported that activation of the TGF‐β signaling enhances the abnormal lymphangiogenesis as well as neoangiogenesis in PM during the progression of peritoneal fibrosis [12–14].

SIRT1 is one of the most extensively studied members of an enzymatic protein family consisting of nicotinamide adenine dinucleotide (NAD^+^)-dependent deacetylases and adenosine diphosphate ribosyltransferases [15]. Accumulating evidence has demonstrated that SIRT1 participates in removing the lysine residues of abundant crucial nuclear factors including p53, p300 and p27, and thus modulating multiple biological processes such as apoptosis, inflammation, stress resistance and senescence [16, 17]. The regulatory role of SIRT1 to TGF‐β signaling has also been revealed recently, and it is reported that SIRT1 inhibits the TGF‐β/Smad3 pathway to attenuate renal fibrosis [18]. However, the regulatory function of SIRT1 on peritoneal fibrosis is largely unclear.

Given the enhancement of TGF‐β to peritoneal fibrosis and the inhibitory function of SIRT1 to TGF‐β signaling, we investigated their connection in peritoneal fibrosis. In this research, we identified decreased SIRT1 expression in PM post PD. We reported that SIRT1 knockout exacerbated peritoneal fibrosis both in vivo and vitro and upregulation of SIRT1 efficiently ameliorated peritoneal fibrosis by inhibiting the secretion of protein matrix induced by TGF‐β signaling.

## Methods

### Animals

Adult C57BL/6 mice (wild type) and myeloid-specific SIRT1 knockout (SIRT1^−/−^) mice weighing between 20 and 30 g were obtained from Beijing Charles River Co., Ltd. All mice used in this research were cultured in the virus/antigen-free system with permanent humidity and constant temperature and free to get the pathogen-free food and water. Animal studies were approved by the ethics committee of Henan Provincial Hospital of Traditional Chinese Medicine.

### Animal model

3 ml of 4.25% glucose dialysis solution (ThermoFisher, Waltham, USA) were administrated by daily intraperitoneal injection for chosen mice to simulate the PD treatment and induce peritoneal fibrosis for 28 days [19]. After 14 days of infusion, the C57BL/6 mice (wild type) and the SIRT1 knockout (SIRT1^−/−^) mice were sacrificed to obtain their peritoneal tissues, including the anterior abdominal wall and omentum, for subsequent analyses. 4% paraformaldehyde (ThermoFisher, Waltham, MA USA) were utilized to fix the collected tissues immediately and all samples were kept at − 80 °C.

### Peritoneal equilibration test (PET)

Modified PET was performed to evaluate the peritoneal permeability before the mice were sacrificed. Blood samples and dialysate were collected at 0 and 120 min of dwell time after intraperitoneal injection of PD fluid. The peritoneal permeability was evaluated by the dialysate-to-plasma (D/P) ratio of blood urea nitrogen (BUN) and the absorption of glucose from the dialysate (D/D0).

### Cell lines and cell culture

The immortalized human pleural mesothelial cell line Met‐5A used in this study were purchased from Beijing Fenghui Biotechnology Co., Ltd. The Medium 199 (Gibco, Grand Island, NY) with 10% fetal bovine serum (Gibco) and 100 U/ml penicillin (Gibco) were used to culture Met-5A cells in an incubator with 100% humidity and 5% carbon dioxide at 37 ℃.

### Cell transfection and treatment

The pHBAd, pHBAd‐SIRT1 and pHBAd‐SIRT1 short hairpin RNA (shRNA) vectors were designed and purchased from the SuZhou GiMa Gene Biotech Co., Ltd (Suzhou, China). 293 T cells were used for adenovirus amplification and packaging. 1 ml cell-free medium containing adenovirus was added into the cell culture, respectively. And the infected Met-5A cells were then cultured in the incubator with 100% humidity and 5% carbon dioxide at 37 ℃ for 48 h before changing the medium. TGF‐β (Gibco) dissolved in phosphate buffered saline (PBS, Gibco) with a concentration of 10 ng/ml were used to treat the Met-5A cells for 48 h for further analysis, and PBS solution were used as the negative control.

### QRT-PCR assay

The total RNA of Met-5A cells and mouse peritoneal tissues were extracted respectively using a TRIzol Kit (ThermoFisher) under the instruction. Then, a SuperScript IV Kit (ThermoFisher) was used to reversely transcribe the mRNA of each sample to cDNA for further experiment. RNA levels of *Sirt1*, *Col1a1*, *Fn*, *α-Sma* and *Snail* were examined by real-time PCR mix and a S1000 PCR Thermal cycler was used to detect the system. *β-actin* served as loading control.

The primers were as follows:

*SIRT1* primer (human).

F: TGTGTCATAGGTTAGGTGGTGA

R: AGCCAATTCTTTTTGTGTTCGTG

*Sirt1* primer (mouse).

F: CAGCCGTCTCTGTGTCACAAA

R: AGCCAATTCTTTTTGTGTTCGTG

*COL1A1* primer (human).

F: ATCAACCGGAGGAATTTCCGT

R: CACCAGGACGACCAGGTTTTC

*Col1a1* primer (mouse).

F: CTGGCGGTTCAGGTCCAAT

R: TTCCAGGCAATCCACGAGC

*FN* primer (human).

F: AGGAAGCCGAGGTTTTAACTG

R: AGGACGCTCATAAGTGTCACC

*Fn* primer (mouse).

F: TTCAAGTGTGATCCCCATGAAG

R: CAGGTCTACGGCAGTTGTCA

*α-SMA* primer (human).

F: GCGTGCGGCTCTACTACATC

R: GCACATTCGGGTCAACTGGTA

*α-Sma* primer (mouse).

F: TCTCCCCGAATCCGATGTCC

R: GCTGGTTCAGCTCGTAGTAGG

*SNAIL* primer (human).

F: CCAGACCCACTCAGATGTCAAG

R: GGGCAGGTATGGAGAGGAAGA

*Snail* primer (mouse).

F: GCACTGTGATGCCCAGGCTA

R: CCTTGCCACAGATCTTGCAGAC

*β-ACTIN* primer (human).

F: TGATGATATCGCCGCGCTC

R: CCATCACGCCCTGGTGC

*β-actin* primer (mouse).

F: TGATGATATCGCCGCGCTC

R: CCATCACGCCCTGGTGC

### Western blot assay

RIPA buffer was used to digest the Met-5A cells and mice peritoneal tissues from the plates and 2 × Loading buffer was used to extract proteins from each sample. A Bicinchoninic Acid protein assay kit (ThermoFisher) was then used to quantify the concentrations of proteins for all samples. A 12% SDS-PAGE was used to separate different proteins in these samples with a voltage of 140 V. And the proteins in the SDS-PAGE were then transferred onto a polyvinylidene difluoride membrane (ThermoFisher) with an electric current of 300 mA. The membrane was co-incubated with anti-SIRT1 and anti-β-actin (1:1000, Abcam, Shanghai, China) at 37 ℃ for 1 h and washed by PBS buffer for 3 times. Then, goat anti‑rabbit IgG (1:5000, Abcam) was incubated with the membrane at 37 ℃ for 1 h and an ECL Western Blotting Substrate kit (Abcam) was used for chemiluminescence imaging.

### Statistical analysis

All experiments in this research were repeated independently at least three times for the accuracy of the data, which were represented as mean ± standard deviation (SD). The GraphPad Prism v7.0 software (GraphPad Software, Inc., La Jolla, USA) was used to analyze the raw data and conduct curves and histogram. Student’s test followed by Mann Whitney test, one-way ANOVA followed by Dunn's multiple comparisons test or two-way ANOVA followed by Sidak's multiple comparisons test were performed to analyze the data from paired groups statistically. P < 0.05 was considered as significant difference in this research.

## Results

### SIRT1 was decreased in the peritoneal omentum tissues of wild type mice post PD

**Fig. 1:**
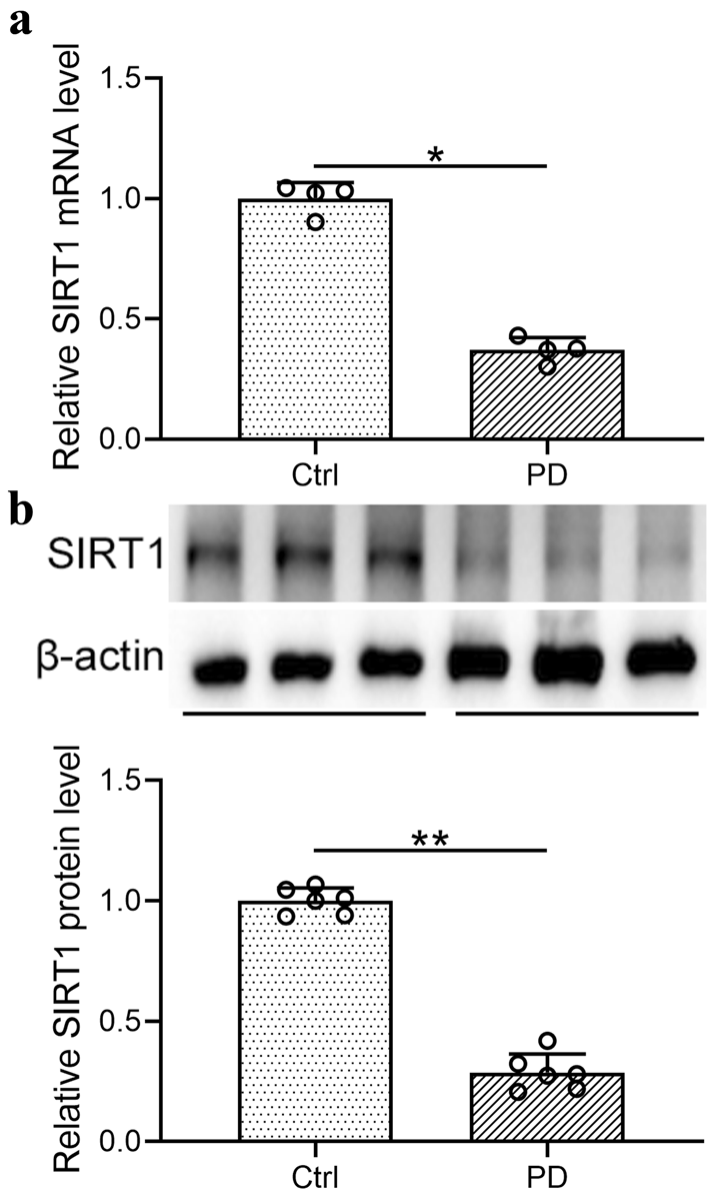
SIRT1 was decreased in the peritoneal omentum tissues of wild-type mice under peritoneal dialysis. **a** Relative SIRT1 mRNA levels were examined by qRT-PCR and normalized to control group. **b** SIRT1 protein expressions were analyzed by Western blotting. β-actin was employed as a loading control. Data are presented as mean ± SD. n = 4 and 6 for each group. *p < 0.05, **p < 0.01 between the indicated groups

To demonstrate the impact of PD procedure and bioincompatible PD solutions to the endogenous SIRT1 expression level of model mice, the peritoneal anterior abdominal wall and omentum were collected from wild type mice, and qRT-PCR and Western blot assays were performed. As shown in Fig. 1a, the *Sirt1* mRNA level in the omentum of wild type mice decreased significantly after the PD treatment. Similarly, the protein level of SIRT1 was also down-regulated post PD (Fig. 1b). Taken together, this figure demonstrated that Sirt1 was decreased in the peritoneal omentum tissues of wild type mice under peritoneal dialysis.

### *The degree of peritoneal fibrosis post PD was exacerbated in SIRT1*^*−/−*^* mice*

**Fig. 2:**
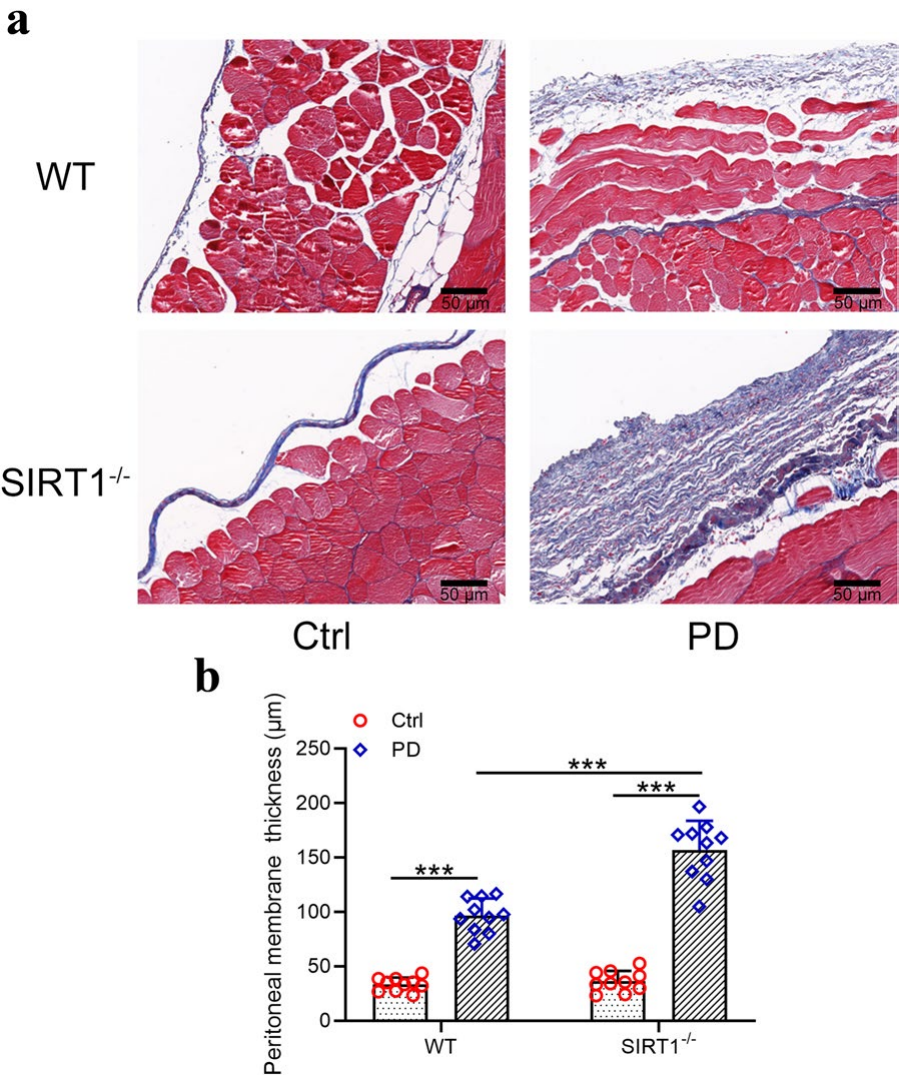
Exacerbated peritoneal fibrosis from SIRT1-/- mice under peritoneal dialysis. **a** Representative Masson’s trichrome staining of the parietal peritoneum in the indicated groups. bar: 50 μm. **b** Mean peritoneal membrane thickness of the submesothelial compact zone in the indicated groups. Data are presented as mean ± SD. n = 10 for each group. ***p < 0.001 between the indicated groups

To further investigate the role of SIRT1 in the development of peritoneal fibrosis induced by PD, the peritoneum of SIRT1^−/−^ mice was collected after the treatment of PD and Masson’s trichrome staining was performed. As shown in Fig. 2a, the protein collagen increased dramatically in the PM of SIRT1^−/−^ mice compared to the control group. The thickness of PM was also measured and as shown in Fig. 2b, PD treatment induced the thickening of PM both in wild type mice and SIRT1^−/−^ mice, but the peritoneal thickness increased by a larger extent in SIRT1^−/−^ mice than wild type mice.

### *The functional failure of peritoneum was exacerbated in SIRT1*^*−/−*^* mice*

**Fig. 3:**
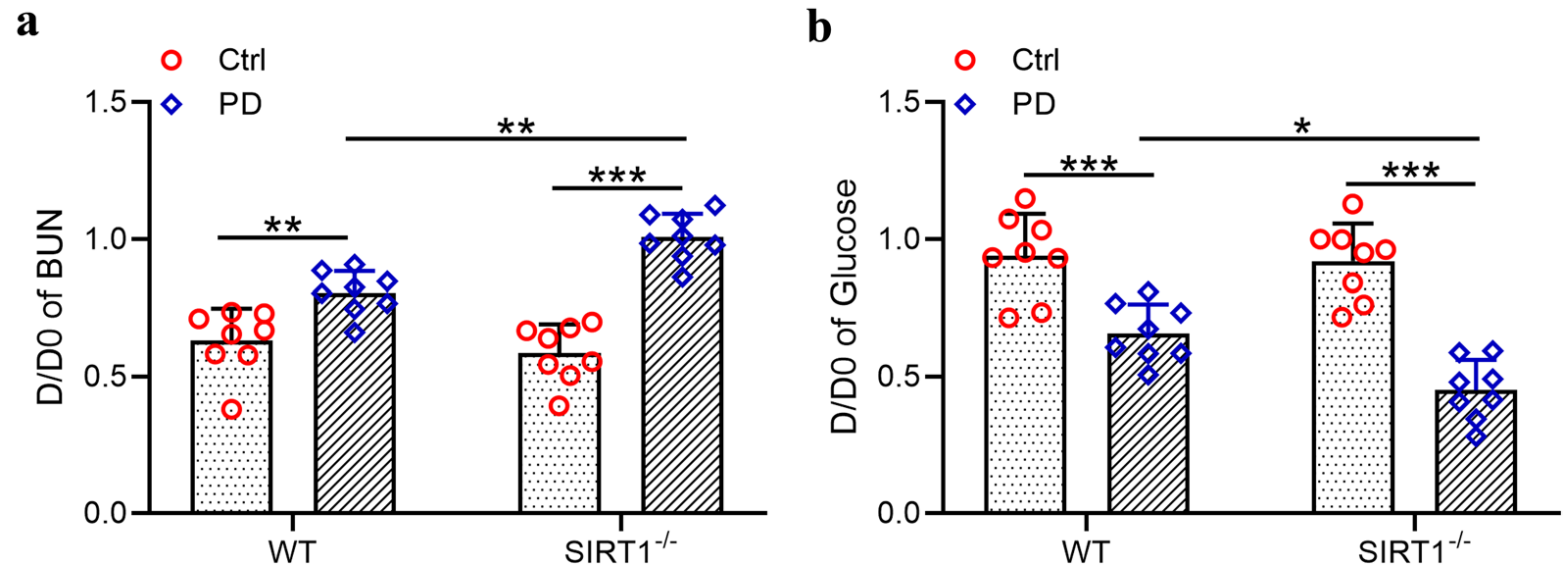
Exacerbated peritoneal functional injury from SIRT1-/- mice under peritoneal dialysis. PET-BUN (**a**) and PET-glucose (**b**) analysis of peritoneal function. Data are presented as mean ± SD. n = 8 for each group. *p < 0.05, **p < 0.01 and ***p < 0.001 between the indicated groups

SIRT1 knockdown enhanced the morphologic alteration of PM induced by peritoneal fibrosis in vivo. However, the impact of SIRT1 knockdown to the function of PM during the progression of peritoneal fibrosis remained unclear. In Fig. 3, the dialysate/plasma (D/P) ratio of BUN and glucose was analyzed for each mouse. The D/D0 of BUN increased both in wild type and SIRT1^−/−^ mice (Fig. 3a), while the D/D0 of glucose decreased both in wild type and SIRT1^−/−^ mice (Fig. 3b). However, there was no significant difference in the altering amplitude of BUN and glucose between the wild type and SIRT1^−/−^ group, indicating that the impact of SIRT1 on the function of PM during peritoneal fibrosis was not crucial.

### *Knockdown of SIRT1 upregulated the expression of matrix proteins during peritoneal fibrosis *in vivo

**Fig. 4:**
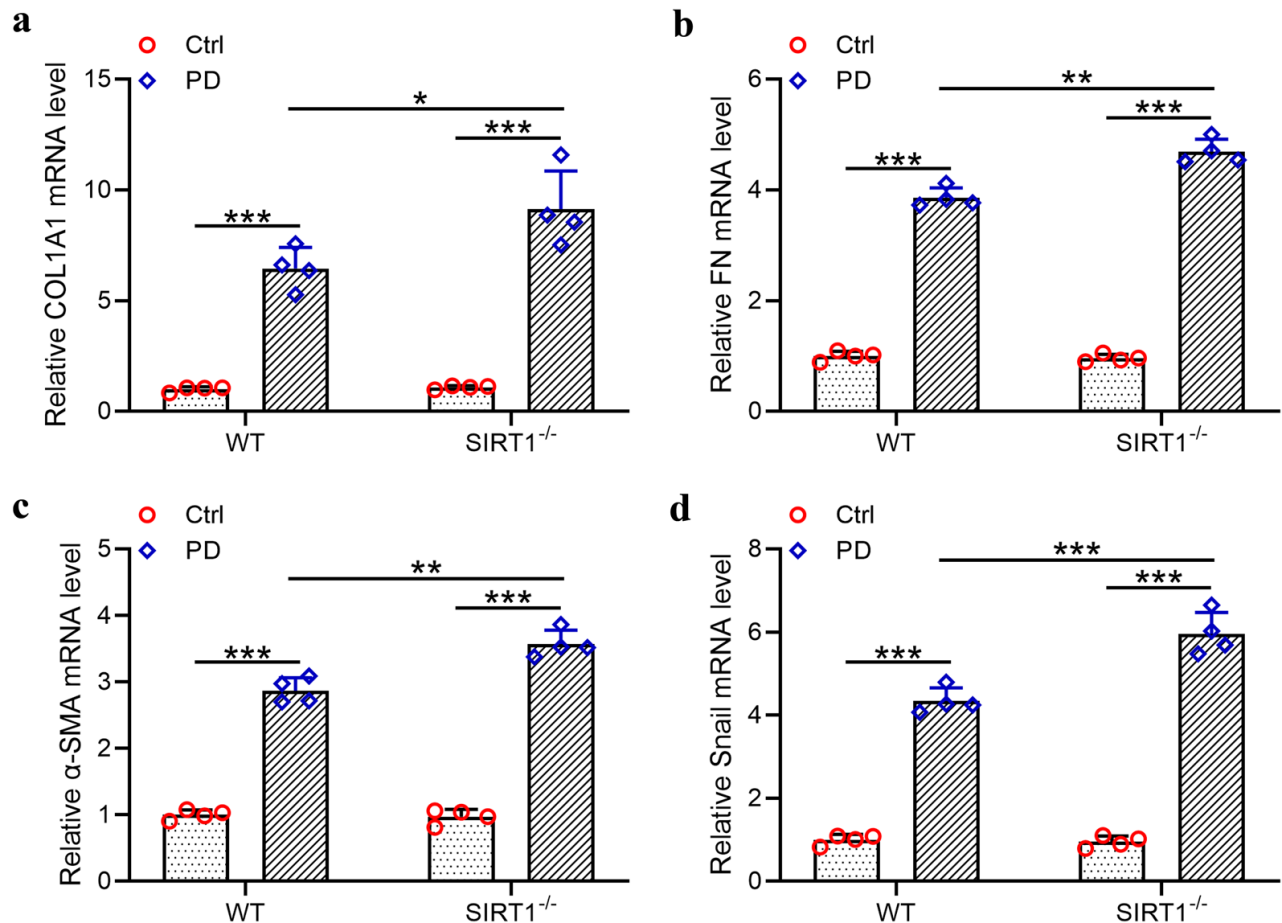
Exacerbated peritoneal fibrosis from SIRT1-/- mice under peritoneal dialysis. Quantitative real time‐PCR was used to measure the mRNA levels of COL1A1 (**a**), FN (**b**), α-SMA (**c**) and Snail (**d**) in the peritoneal omentum tissues from indicated groups. Data are presented as mean ± SD. n = 4 for each group. *p < 0.05, **p < 0.01, ***p < 0.001 between the indicated groups

Knockdown of SIRT1 induced the thickening of PM post PD treatment in vivo. To demonstrate the underlying mechanisms of this process, the expression levels of four extracellular matrix proteins including Col1a1, Fn, α-Sma and Snail in the peritoneal omentum tissues of SIRT1^−/−^ mice were analyzed by qRT-PCR assays. As shown in Fig. 4a–d, the four extracellular matrix proteins all increased after the PD treatment in both wild type and SIRT1^−/−^ mice. And the peritoneal omentum tissues of SIRT1^−/−^ mice produced more extracellular matrix proteins post PD therapy. Thus, the upregulation of extracellular matrix proteins including COL1A1, FN, α-SMA and Snail induced by PD and SIRT1 knockdown contributed to the thickening of PM.

### *PD induced exacerbated inflammatory response in SIRT1*^*−/−*^* mice*

**Fig. 5:**
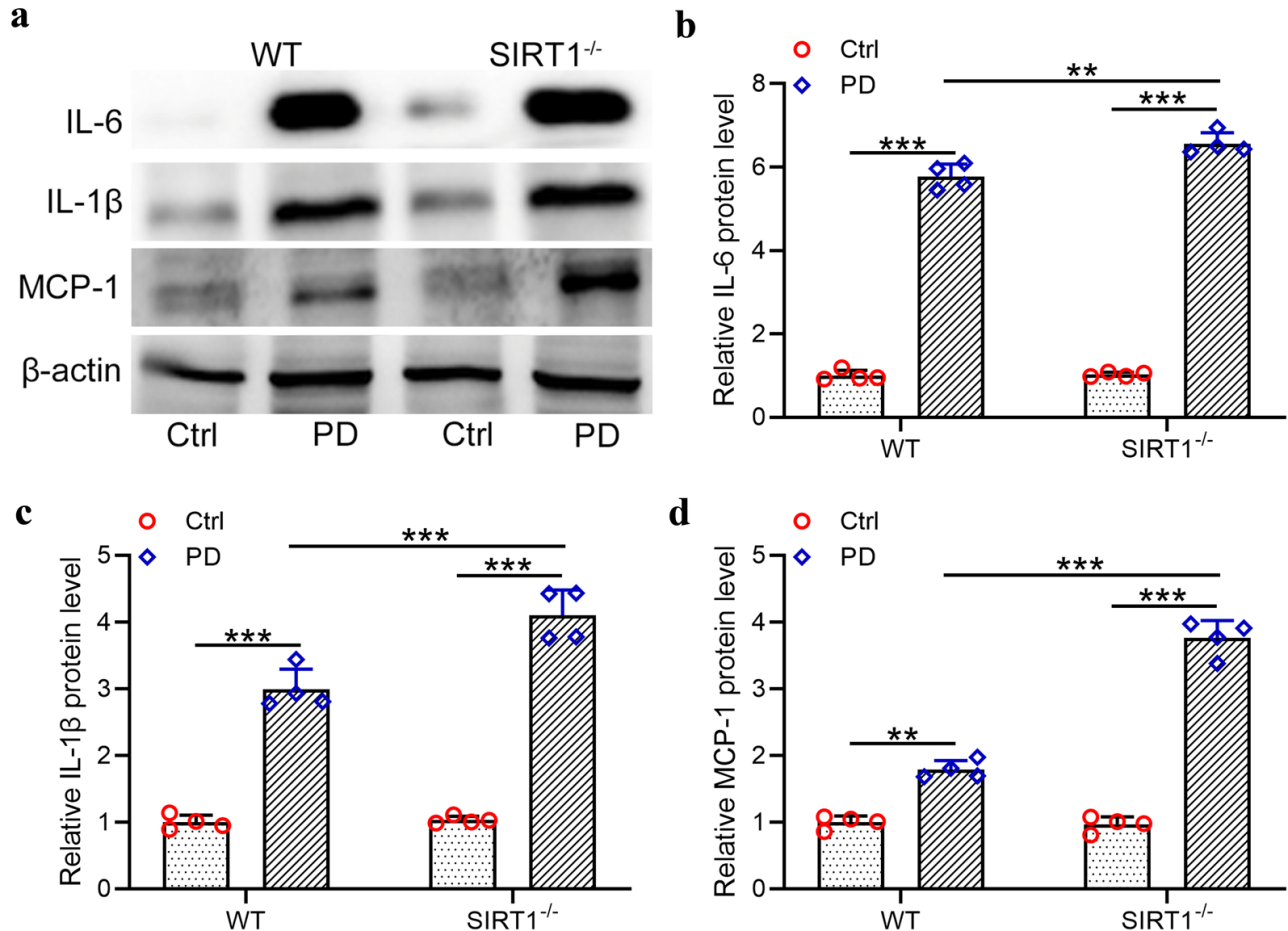
Exacerbated inflammatory response from SIRT1-/- mice under peritoneal dialysis. **a** Western blotting was used to measure the protein expressions of IL-6, IL-1β and MCP-1 in the peritoneal omentum tissues from indicated groups. The relative expressions were normalized to control in WT (**b**–**d**). Data are presented as mean ± SD. n = 4 for each group. **p < 0.01, ***p < 0.001 between the indicated groups

To further demonstrate the impact of SIRT1 deficiency on the inflammatory response in mice post PD, Western blot assay was performed and the protein levels of IL-6, IL-1β and MCP-1 were evaluated. As shown in Fig. 5a and b, PD treatment induced a significant increase in IL-6 protein level in the mouse peritoneum compared with the control group. Moreover, SIRT1 knockout caused a further increase in IL-6 protein level. Similarly, SIRT1 deficiency also upregulated the protein levels of IL-1β (Fig. 5c) and MCP-1 (Fig. 5d) in the mouse peritoneum. These data suggested that knockout of SIRT1 enhanced the inflammation response in the peritoneum of PD-treated mice.

### SIRT1 deficiency enhanced the TGF-β/SMAD3 pathway involved in PD-induced peritoneal fibrosis

**Fig. 6:**
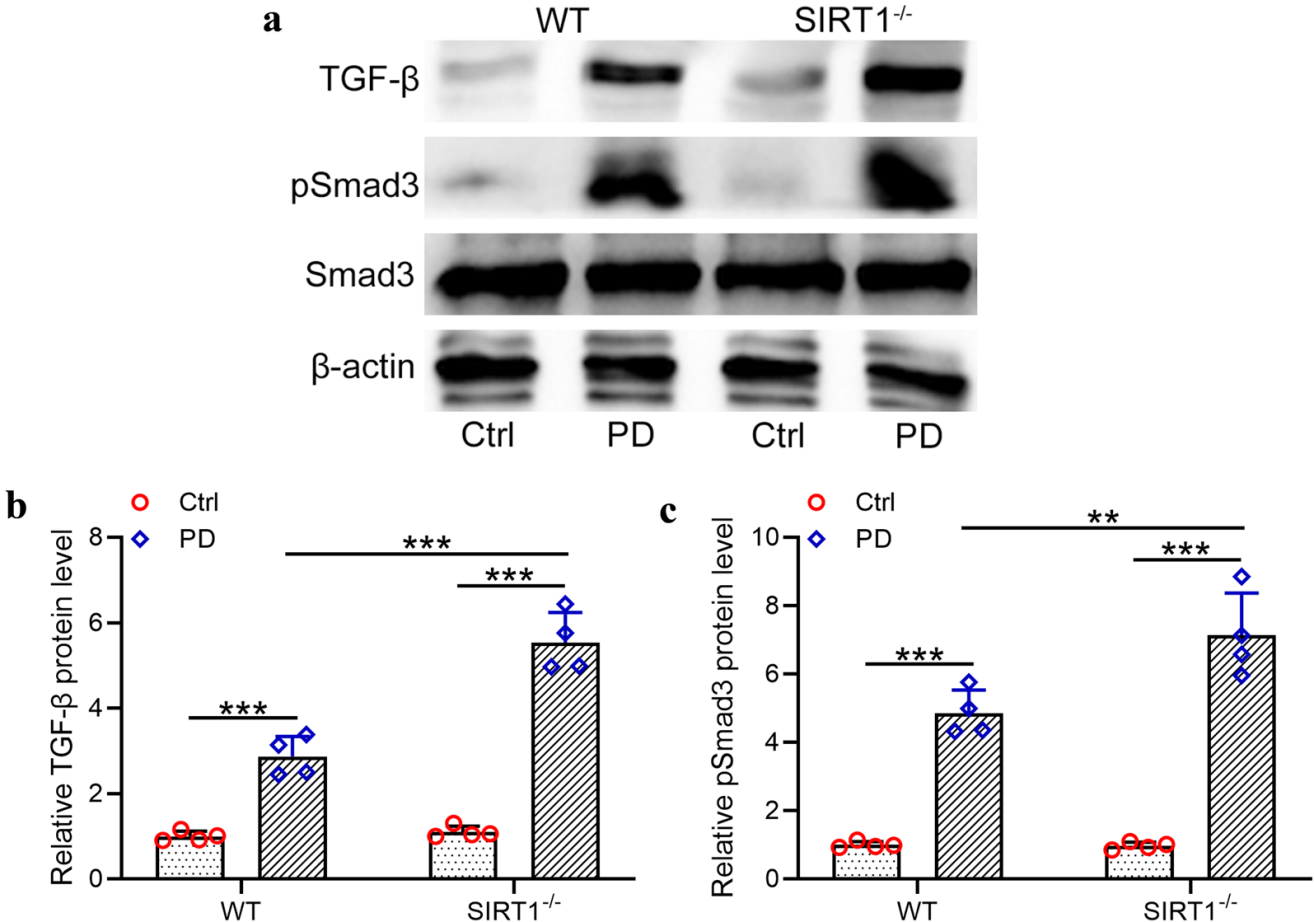
TGF-β/Smad3 pathway was involved in peritoneal dialysis induced peritoneal fibrosis. **a** Western blotting was used to measure the protein expressions of TGF-β, pSmad3 and Smad3 in the peritoneal omentum tissues from indicated groups. The relative expressions were normalized to control in WT (**b**, **c**). Data are presented as mean ± SD. n = 4 for each group. **p < 0.01, ***p < 0.001 between the indicated groups

Western blot assay was performed to evaluate the impact of SIRT1 deficiency on the TGF-β/SMAD3 pathway in the PD-treated peritoneum. As shown in Fig. 6a, b, PD treatment induced a significant upregulation of TGF-β protein level in the mouse peritoneum compared with the wild type group. Moreover, SIRT1 knockout further increased TGF-β protein level. Similarly, SIRT1 deficiency also upregulated the phosphorylation of SMAD3 (Fig. 6c) in the mouse peritoneum, indicating that SIRT1 deficiency further activated the TGF-β/SMAD3 signaling pathway which was activated by PD treatment.

### SIRT1 was downregulated in TGF‐β stimulated Met‐5A cells

**Fig. 7:**
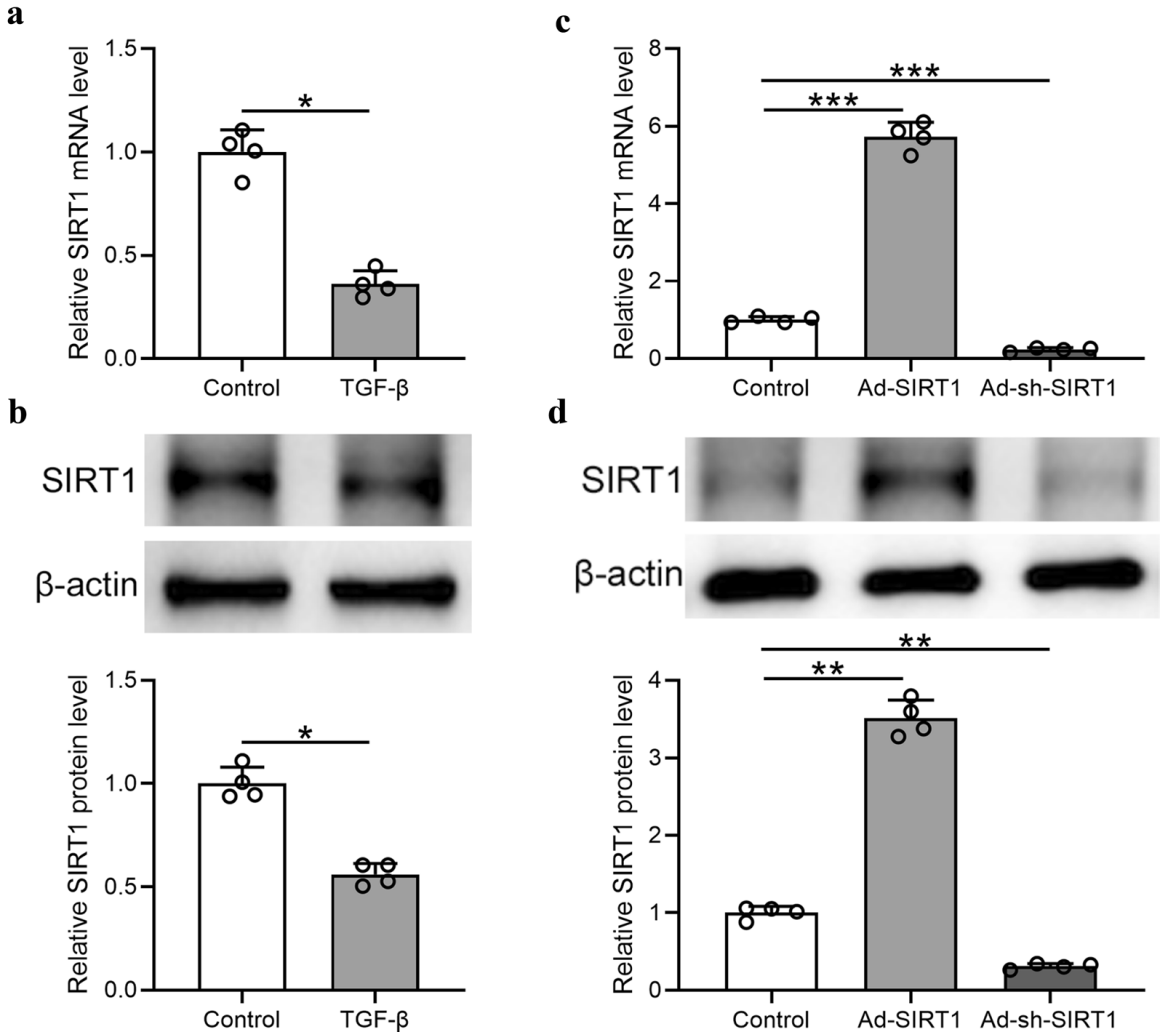
SIRT1 was downregulated in TGF‐β stimulated Met‐5A cells and Adenovirus transfection of SIRT1. **a** SIRT1 mRNA expression assessed by qRT‐PCR in Met‐5A cells exposed to control conditions (unstimulated) or TGF‐β1 for 48 h. **b** SIRT1 protein level assessed by Western blotting under identical conditions and the relative expressions were normalized to control. **c** Met‐5A cells were transfected with Ad‐sh‐SIRT1, or Ad‐SIRT1 for 48 h. Non-transfected cells were included as control. SIRT1 mRNA expression assessed by qRT‐PCR. (D) SIRT1 protein level assessed by Western blotting after transfection and the relative expressions were normalized to control. Data are presented as mean ± SD. n = 4 for each group. *p < 0.05, **p < 0.01, ***p < 0.001 between the indicated groups

TGF‐β treatment of human Met-5A cells induced the down-regulation of SIRT1. The mRNA and protein levels of SIRT1 in Met-5A cells were shown in Fig. 7a, b, both of which were decreased by TGF‐β treatment.

### Adenovirus targeting SIRT1

To regulate the expression level of SIRT1 in Met-5A cells, Ad‐sh‐SIRT1 and Ad‐SIRT1 were packaged and amplified. Ad‐sh‐SIRT1 transfection decreased SIRT1 mRNA in Met-5A cells and Ad‐SIRT1 had the opposite function (Fig. 7c). Besides, the adenovirus also regulated the protein levels of SIRT1 (Fig. 7d) with the same trend as mRNA level.

### Overexpression of SIRT1 inhibited TGF-β1-induced epithelial-mesenchymal transition in Met‐5A cells

**Fig. 8:**
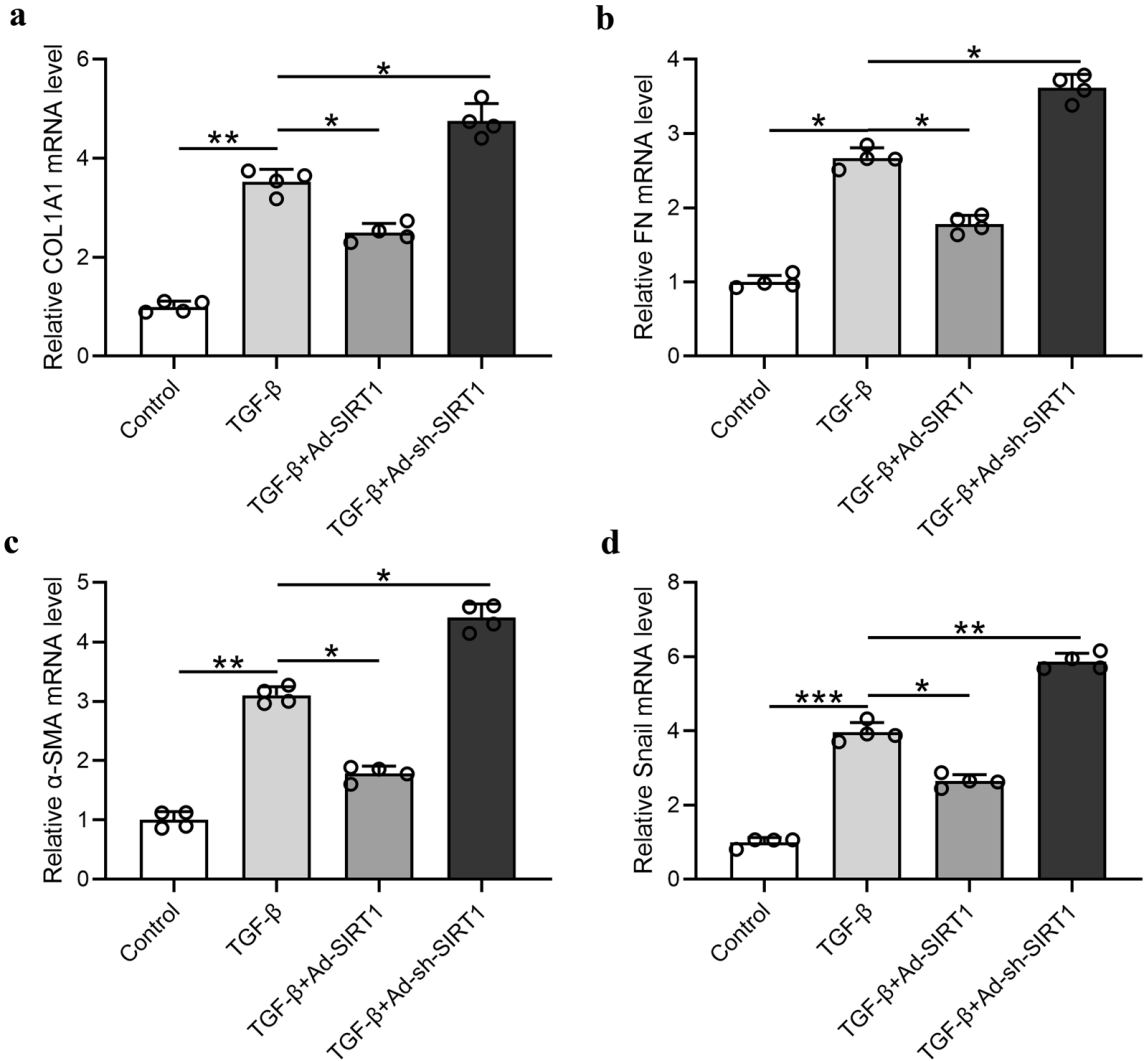
Overexpression of SIRT1 inhibited TGF-β1-induced epithelial-mesenchymal transition in Met‐5A cells. Quantitative real time‐PCR was used to measure the mRNA levels of COL1A1 (**a**), FN (**b**), α-SMA (**c**) and Snail (**d**) in the indicated conditions. Data are presented as mean ± SD. n = 4 for each group. *p < 0.05, **p < 0.01, ***p < 0.001 between the indicated groups

To demonstrate the regulatory role of SIRT1 to TGF-β1-induced peritoneal fibrosis, the Ad‐sh‐SIRT1 and Ad‐SIRT1 were transfected respectively into Met-5A cells, which were then treated with TGF-β. As shown in Fig. 8a–d, the four extracellular matrix proteins showed a similar trend with different stimulation. TGF-β stimulation itself significantly upregulated all four proteins, while overexpression of SIRT1 in vitro dramatically inhibited their expression. Taken together, overexpression of SIRT1 inhibited TGF-β1-induced epithelial-mesenchymal transition in Met‐5A cells.

### Overexpression of SIRT1 inhibited TGF-β1-induced inflammatory response in Met‐5A cells

**Fig. 9:**
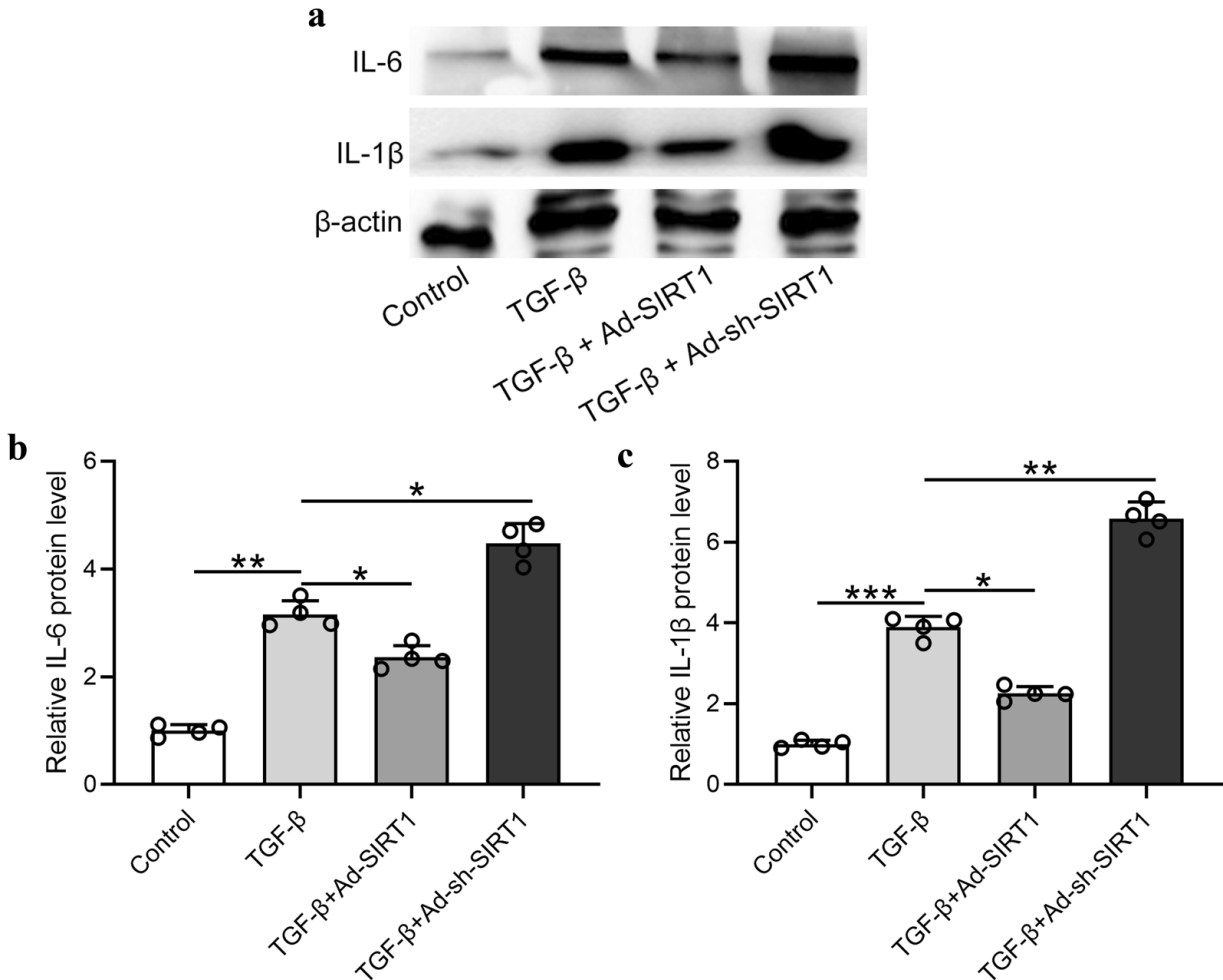
Overexpression of SIRT1 inhibited TGF-β1-induced inflammatory response in Met‐5A cells. **a**–**c** Western blotting was used to measure the protein expressions of IL-6 and IL-1β, in the indicated conditions. Data are presented as mean ± SD. n = 4 for each group. *p < 0.05, **p < 0.01, ***p < 0.001 between the indicated groups

The protein levels of IL-6 and IL-1β in the TGF-β1-stimulated Met-5A cells were evaluated by Western blot assay. As presented in Fig. 9a–c, TGF-β upregulated both IL-6 and IL-1β in Met‐5A cells, while overexpression of SIRT1 downregulated the protein levels of IL-6 and IL-1β in TGF-β stimulated Met‐5A cells. On the contrary, knockdown of SIRT1 in TGF-β-stimulated Met‐5A cells significantly upregulated IL-6 and IL-1β, suggesting that SIRT1 played a crucial role in the inhibition of peritoneal inflammation.

## Discussion

Peritoneal fibrosis is characterized by reduced mesothelial cells, neovascularization and continuous thickening of the sub-peritoneal mesothelial dense layer with increased fibroblasts [8, 20]. The peritoneal dialysate has been regarded as the most important contributing factor for peritoneal fibrosis [21]. Overexpression of inflammatory factors during this process prompts the excessive deposition of extracellular matrix and eventually leads to peritoneal fibrosis [22]. Previous studies have revealed that the signaling pathways involved in peritoneal fibrosis are very complex, mainly including the cannabinoid signaling pathway, oxidative stress signaling pathway, TGF-β signaling pathway, NF-kB signaling pathway, Sonic Hedgehog signaling pathway, etc. [23–25].

In our previous study, we have explained the important relationship between SIRT1 and the development of peritoneal fibrosis. We overexpressed SIRT1 in hUCMSCs, which was then used to treat the PD model mice. We found that this SIRT1-modified hUCMSCs ameliorated experimental peritoneal fibrosis by inhibiting the TGF-β/Smad3 pathway. Therefore, we directly knocked out SIRT1 in mice in this study to further reveal its regulatory mechanism in the development of peritoneal fibrosis.

TGF-β plays an important role in the occurrence and progression of peritoneal fibrosis. Accumulating evidence has demonstrated that TGF-β is a central mediator for the progression of peritoneal fibrosis, which regulates intracellular signaling through the TGF-β/Smad pathway and participates in the entire process of fibrosis [26]. Phosphorylated TGF-βR I induced the phosphorylation of SMAD2 and SMAD3, thus mediating the gene transcription, promoting collagen production and stimulating the secretion of extracellular matrix of fibroblasts located at the sub-mesothelium layer of PM [27]. In addition, TGF-β can up-regulate the production of the enzymes that inhibit the degradation of extracellular matrix and reduce the production of extracellular matrix degradation enzymes, thereby inducing the accumulation of extracellular matrix in the sub-mesothelium layer of PM [11]. During the progression of peritoneal fibrosis, the extracellular matrix integrin signal induces the expression of MCP-1, leading to the infiltration of macrophages at the site of inflammation [28–30].

In this research, we reported that SIRT1 knockout also upregulated the protein levels of TGF-β (p < 0.001) and pSMAD3 (p < 0.01) in PD mice, indicating that SIRT1 deficiency activated the TGF-β/SMAD3 pathway and enhanced peritoneal fibrosis. Moreover, SIRT1 knockout significantly upregulated IL-6 (p < 0.01), IL-1β (p < 0.001) and MCP-1 (p < 0.001) in PD mouse model, suggesting that SIRT1 deficiency promoted the PD-induced inflammation. Moreover, we reported that overexpression of SIRT1 down-regulated the protein levels of IL-6 (p < 0.05) and IL-1β (p < 0.05) in TGF-β stimulated Met‐5A cells. On the contrary, SIRT1 knockdown in TGF-β stimulated Met‐5A cells significantly upregulated IL-6 (p < 0.05) and IL-1β (p < 0.01), suggesting that SIRT1 played a crucial role in the inhibition of peritoneal inflammation.

According to the different pathogenesis of peritoneal fibrosis, researchers have proposed corresponding treatment methods. For instance, dextran peritoneal dialysate has been used in clinic for patients with peritoneal fibrosis. Dextran peritoneal dialysate is an isotonic peritoneal dialysate with less glucose and low GDPs, which can increase the removal of water and reduce the adverse effects of peritoneal and systemic exposure to hypertonic glucose [31]. Suppressor of cytokine signaling (SOCS) can be used to inhibit cytokine production, down-regulate cytokine-induced CTGF and VEGF expression levels, and thereby improve peritoneal fibrosis and angiogenesis post PD treatment [12]. However, the efficacy of current treatments for peritoneal fibrosis remains widely controversial in academia, and our understanding on the mechanisms of peritoneal fibrosis is quite limited [11]. In this research, we investigated the regulatory function of SIRT1 in the TGF-β signaling induced peritoneal fibrosis. We firstly reported that SIRT1 could inhibit the production of the extracellular matrix proteins both in vivo and in vitro, which was activated by the TGF-β signaling during the development of peritoneal fibrosis. Our discovery could provide a new perspective for the mechanisms underlying the mesothelial-mesenchymal transition during the peritoneal fibrosis progression.

SIRT1 is a mammalian ortholog of SIR2 and is a highly conserved NAD-dependent protein deacetylase [32]. As a key regulatory molecule, SIRT1 plays a vital role in metabolic homeostasis. Previous studies have shown that SIRT1 can suppress inflammatory responses in a variety of cells and tissues [32]. For instance, it has been reported that SIRT1 knockout in hepatocytes aggravates inflammatory response in the liver, and SIRT1 can also participate in liver fibrosis by regulating the TGF-β signaling pathway [33]. Another study then revealed that resveratrol, an activator of Sirt1, attenuated the progress of renal fibrosis both in vitro and in vivo by inhibiting the TGF-β/SMAD3 signaling pathway and reducing the stress caused by reactive oxygen species [18]. In addition, the close relationship between SIRT1 expression in atrial tissue and atrial fibrosis in patients with atrial fibrillation has also been reported. Similar to these former discoveries, our previous study also found that SIRT1 could effectively inhibit peritoneal fibrosis both in vivo and in vitro [34]. Interestingly, although SIRT1 has an important regulatory effect on the function and structure of the peritoneum, knocking down SIRT1 in this study did not seem to significantly affect the ultrafiltration function of the mouse peritoneum. A variety of cell pathways and numerous pro-fibrosis factors are involved in the occurrence and development of peritoneal fibrosis. Among them, TGF-β-mediated trans-differentiation of peritoneal epithelial cells plays a central role. We believe that SIRT1, as an anti-fibrosis factor, plays a role in the anti-fibrosis process mediated by TGF-β/Smad signaling. Indeed, we acknowledge that our understanding of the molecular mechanism by which SIRT1 regulates the balance of pro-fibrotic cytokines and anti-fibrotic cytokines is still limited. We will continue to focus on the myeloid specificity of SIRT1 knockout and study the regulatory effect of SIRT1 on the inflammatory response in future research.

## Conclusion

In conclusion, we reported that SIRT1 ameliorated peritoneal fibrosis both in vivo and in vitro through inhibiting the expression of matrix proteins induced by TGF‐β signaling. We identified decreased expression of SIRT1 in vivo induced by PD-like treatment, which could be a possible factor for the development of peritoneal fibrosis. Our discovery could provide a new perspective to explain the mechanisms underlying the peritoneal dysfunction post PD treatment.

## Data Availability

All data generated or analyzed during this study are included in this published article.

## Abbreviations

PD: Peritoneal dialysis
TGF‐β: Transforming growth factor‐β
MCs: Mesothelial cells
SIRT1: Sirtuin1
PM: Peritoneal membrane
GDP: Glucose degradation products
AGEs: Advanced glycation end products
NAD+: Nicotinamide adenine dinucleotide
SIRT1−/−: SIRT1 knockout
PET: Peritoneal equilibration test
D/P: Dialysate-to-plasma
BUN: Blood urea nitrogen
PBS: Phosphate buffered saline

## References

[1] Alatab S, Najafi I, Tabatabaei-Malazy O, Pourmand G, Ahmadbeigi N (2019). Strategies for prevention and treatment of peritoneal fibrosis: a scientometric study. Int J Prev Med.

[2] Ashizawa N, Miyazaki T, Abe S, Takazono T, Saijo T, Obata Y, Shimamura S, Yamamoto K, Imamura Y, Koji T (2019). Evaluation of *Candida peritonitis* with underlying peritoneal fibrosis and efficacy of micafungin in murine models of intra-abdominal candidiasis. Sci Rep.

[3] Asifullah K, Zhou Z, He W, Gao K, Khan MW, Faisal R, Muhammad H, Sun M (2019). CXCR4-receptor-targeted liposomes for the treatment of peritoneal fibrosis. Mol Pharm.

[4] Helmke A, Nordlohne J, Balzer MS, Dong L, Rong S, Hiss M, Shushakova N, Haller H, von Vietinghoff S (2019). CX3CL1-CX3CR1 interaction mediates macrophage-mesothelial cross talk and promotes peritoneal fibrosis. Kidney Int.

[5] Jiang N, Zhang Z, Shao X, Jing R, Wang C, Fang W, Mou S, Ni Z (2020). Blockade of thrombospondin-1 ameliorates high glucose-induced peritoneal fibrosis through downregulation of TGF-beta1/Smad3 signaling pathway. J Cell Physiol.

[6] Nam BY, Park JT, Kwon YE, Lee JP, Jung JH, Kim Y, Kim S, Park J, Um JE, Wu M (2017). Periostin-binding DNA aptamer treatment ameliorates peritoneal dialysis-induced peritoneal fibrosis. Mol Ther Nucleic Acids.

[7] Kunin M, Carmon V, Beckerman P, Dinour D (2019). Effect of peritoneal dialysis on serum fibrosis biomarkers in patients with refractory congestive heart failure. Int J Mol Sci.

[8] Balzer MS (2020). Molecular pathways in peritoneal fibrosis. Cell Signal.

[9] Alatab S, Najafi I, Atlasi R, Pourmand G, Tabatabaei-Malazy O, Ahmadbeigi N (2018). A systematic review of preclinical studies on therapeutic potential of stem cells or stem cells products in peritoneal fibrosis. Minerva Urol Nefrol.

[10] Chen YT, Hsu H, Lin CC, Pan SY, Liu SY, Wu CF, Tsai PZ, Liao CT, Cheng HT, Chiang WC (2020). Inflammatory macrophages switch to CCL17-expressing phenotype and promote peritoneal fibrosis. J Pathol.

[11] Yao Q, Pawlaczyk K, Ayala ER, Styszynski A, Breborowicz A, Heimburger O, Qian JQ, Stenvinkel P, Lindholm B, Axelsson J (2008). The role of the TGF/Smad signaling pathway in peritoneal fibrosis induced by peritoneal dialysis solutions. Nephron Exp Nephrol.

[12] Yoshizawa H, Morishita Y, Watanabe M, Ishibashi K, Muto S, Kusano E, Nagata D (2015). TGF-beta(1)-siRNA delivery with nanoparticles inhibits peritoneal fibrosis. Gene Ther.

[13] Ueno T, Nakashima A, Doi S, Kawamoto T, Honda K, Yokoyama Y, Doi T, Higashi Y, Yorioka N, Kato Y (2013). Mesenchymal stem cells ameliorate experimental peritoneal fibrosis by suppressing inflammation and inhibiting TGF-beta1 signaling. Kidney Int.

[14] Subeq YM, Ke CY, Lin NT, Lee CJ, Chiu YH, Hsu BG (2011). Valsartan decreases TGF-beta1 production and protects against chlorhexidine digluconate-induced liver peritoneal fibrosis in rats. Cytokine.

[15] Qian W, Cai X, Qian Q (2020). Sirt1 antisense long non-coding RNA attenuates pulmonary fibrosis through sirt1-mediated epithelial-mesenchymal transition. Aging (Albany NY).

[16] Lee SJ, Kim SJ, Lee HS, Kwon OS (2019). PKCdelta mediates NF-kappaB inflammatory response and downregulates SIRT1 expression in liver fibrosis. Int J Mol Sci.

[17] Li M, Hong W, Hao C, Li L, Wu D, Shen A, Lu J, Zheng Y, Li P, Xu Y (2018). SIRT1 antagonizes liver fibrosis by blocking hepatic stellate cell activation in mice. FASEB J.

[18] Huang XZ, Wen D, Zhang M, Xie Q, Ma L, Guan Y, Ren Y, Chen J, Hao CM (2014). Sirt1 activation ameliorates renal fibrosis by inhibiting the TGF-beta/Smad3 pathway. J Cell Biochem.

[19] Zhang Y, Huang Q, Chen Y, Peng X, Wang Y, Li S, Wu J, Luo C, Gong W, Yin B (2020). Parthenolide, an NF-kappaB inhibitor, alleviates peritoneal fibrosis by suppressing the TGF-beta/Smad pathway. Int Immunopharmacol.

[20] Devuyst O, Margetts PJ, Topley N (2010). The pathophysiology of the peritoneal membrane. J Am Soc Nephrol.

[21] Zhang F, Liu H, Liu F, Peng Y, Chen M, Liu Y, Chen G (2013). New insights into the pathogenesis and treatment of peritoneal fibrosis: a potential role of Wnt/beta-catenin induced epithelial to mesenchymal transition and stem cells for therapy. Med Hypotheses.

[22] Li J, Li SX, Gao XH, Zhao LF, Du J, Wang TY, Wang L, Zhang J, Wang HY, Dong R, Guo ZY (2019). HIF1A and VEGF regulate each other by competing endogenous RNA mechanism and involve in the pathogenesis of peritoneal fibrosis. Pathol Res Pract.

[23] Guo Y, Wang L, Gou R, Wang Y, Shi X, Pang X, Tang L (2020). SIRT1-modified human umbilical cord mesenchymal stem cells ameliorate experimental peritoneal fibrosis by inhibiting the TGF-beta/Smad3 pathway. Stem Cell Res Ther.

[24] Strippoli R, Moreno-Vicente R, Battistelli C, Cicchini C, Noce V, Amicone L, Marchetti A, Del Pozo MA, Tripodi M (2016). Molecular mechanisms underlying peritoneal EMT and fibrosis. Stem Cells Int.

[25] Yang CY, Chau YP, Chen A, Lee OK, Tarng DC, Yang AH (2017). Targeting cannabinoid signaling for peritoneal dialysis-induced oxidative stress and fibrosis. World J Nephrol.

[26] Zhou Q, Yang M, Lan H, Yu X (2013). miR-30a negatively regulates TGF-beta1-induced epithelial-mesenchymal transition and peritoneal fibrosis by targeting Snai1. Am J Pathol.

[27] Derynck R, Zhang YE (2003). Smad-dependent and Smad-independent pathways in TGF-beta family signalling. Nature.

[28] Balzer MS, Helmke A, Ackermann M, Casper J, Dong L, Hiss M, Kiyan Y, Rong S, Timrott K, von Vietinghoff S (2019). Protein kinase C beta deficiency increases glucose-mediated peritoneal damage via M1 macrophage polarization and up-regulation of mesothelial protein kinase C alpha. Nephrol Dial Transplant.

[29] Meng XM, Huang XR, Xiao J, Chung AC, Qin W, Chen HY, Lan HY (2012). Disruption of Smad4 impairs TGF-beta/Smad3 and Smad7 transcriptional regulation during renal inflammation and fibrosis in vivo and in vitro. Kidney Int.

[30] Phillips AO, Fraser DJ (2010). BMP-7 stops TGF-{beta} in peritoneal fibrosis. Nephrol Dial Transplant.

[31] Stamm SJ, Doctor J, Rose R, Isbister J, Hickman R (1966). Peritoneal dialysis in the treatment of cystic fibrosis with congestive heart failure. Clin Pediatr (Phila).

[32] Zerr P, Palumbo-Zerr K, Huang J, Tomcik M, Sumova B, Distler O, Schett G, Distler JH (2016). Sirt1 regulates canonical TGF-beta signalling to control fibroblast activation and tissue fibrosis. Ann Rheum Dis.

[33] Sun L, Fan Z, Chen J, Tian W, Li M, Xu H, Wu X, Shao J, Bian Y, Fang M, Xu Y (2016). Corrigendum: transcriptional repression of SIRT1 by protein inhibitor of activated STAT 4 (PIAS4) in hepatic stellate cells contributes to liver fibrosis. Sci Rep.

[34] Han L, Tang Y, Li S, Wu Y, Chen X, Wu Q, Hong K, Li J (2020). Protective mechanism of SIRT1 on Hcy-induced atrial fibrosis mediated by TRPC3. J Cell Mol Med.

